# Development and validation of a prognostic model for acute-on-chronic liver failure

**DOI:** 10.3389/fcimb.2026.1759738

**Published:** 2026-02-09

**Authors:** Xia Zhu, Ming Wang, Yuanji Ma, Libo Yan, Jianhao Li, Hong Peng, Yue Huang, Hong Tang

**Affiliations:** 1 Center of Infectious Diseases, West China Hospital of Sichuan University, Chengdu, China; 2 Laboratory of Infectious and Liver Diseases, Institute of Infectious Diseases, West China Hospital of Sichuan University, Chengdu, China; 3 Department of Infectious Diseases, Guizhou Provincial People’s Hospital, Guizhou, China

**Keywords:** acute-on-chronic liver failure, hepatitis B virus, liver reserve function, machine learning, prognosis

## Abstract

**Background:**

Prognostic assessment in acute-on-chronic liver failure (ACLF), particularly in HBV-endemic regions, remains challenging due to the limited accuracy of conventional models. We aimed to develop and validate a novel, machine learning-based model incorporating liver reserve function to improve individualized prediction of short-term outcomes in HBV-ACLF.

**Methods:**

Baseline demographics, clinical features, laboratory findings, and 90-day follow-up data were retrospectively collected from 496 patients (training/internal subgroups) and 52 patients (external validation) with HBV-ACLF. Twelve machine learning algorithms were systematically evaluated for prognostic performance. The optimal model was established using the LASSO-RF approach, with key variables identified by SHAP values. Model accuracy was assessed by ROC analysis and compared with MELD and CTP scores. An interactive web calculator (https://syx123.shinyapps.io/deploy_shiny/) was developed to facilitate clinical use.

**Results:**

We initially screened 23 potential clinical risk factors for predicting ACLF prognosis. Subsequently, using the LASSO-RF model, 15 key variables were selected for model construction. The final LASSO-RF model achieved an AUC of 0.99 in the training cohort and 0.98 in the validation cohort for predicting 90-day mortality, outperforming conventional scoring systems such as MELD and CTP. To facilitate clinical application, an online tool (https://syx123.shinyapps.io/deploy_shiny/) was developed to provide real-time risk scores and 90-day mortality predictions for individual patients.

**Conclusions:**

Liver reserve function indicators, particularly EHBF and ICG-R15, play a pivotal role in prognosticating HBV-ACLF outcomes. The developed model and its accompanying online tool enable accurate risk stratification and have the potential to guide timely and individualized clinical management.

## Background

Acute-on-chronic liver failure (ACLF) is a syndrome marked by the rapid deterioration of liver cirrhosis and the onset of liver dysfunction, often linked to high short-term mortality rates (Wu et al., 2018; Sarin et al., 2019; Bajaj et al., 2022; Butt and Jalan, 2023; European Association for the Study of the, L, 2023). Research has established viral hepatitis and chronic alcohol abuse as leading causes of ACLF (European Association for the Study of the, L, 2023). In China, approximately 75 million people are living with chronic hepatitis B (CHB), and HBV remains a major precipitating etiology of ACLF (Yan et al., 2025). ACLF mechanisms are largely attributed to intense systemic inflammation, immune dysregulation, metabolic abnormalities, oxidative stress, and impaired mitochondrial function (Moreau et al., 2013; Engelmann et al., 2021). Although recent studies have made significant progress in understanding the pathophysiological mechanisms of ACLF, there remains a lack of specific therapeutic strategies beyond liver transplantation (Otsuka et al., 2007; Demirtas et al., 2021; Li et al., 2024). Practical challenges, such as organ shortages and high costs, further complicate treatment. Therefore, accurate early prognosis for ACLF patients is crucial for optimizing therapeutic decisions and improving liver allocation.

Hepatic functional reserve denotes the liver’s ability to adapt to heightened physiological demands after an injury (Michalopoulos and Bhushan, 2021). This capacity is largely determined by the quantity of viable hepatocytes and the preservation of liver tissue structure, which together indicate the organ’s compensatory potential. The indocyanine green (ICG) clearance rate is a commonly used method for assessing hepatic reserve function (Moody et al., 1974; Manizate et al., 2010; Levesque et al., 2016; Lu et al., 2022; Caimano et al., 2024). Research indicates that the ICG retention rate at 15 minutes (ICG-R15) offers superior predictive accuracy for postoperative liver failure in patients with hepatocellular carcinoma (HCC) compared to the Child-Turcotte-Pugh (CTP) score and the Model for End-Stage Liver Disease (MELD) score, both of which are commonly utilized to evaluate liver disease severity and prognosis (Wang et al., 2018; Zhang et al., 2022). Moreover, liver reserve-related indicators such as ICG-R15 and effective hepatic blood flow (EHBF) exhibit high sensitivity and specificity in predicting HBV-ACLF (Chen et al., 2021). The combined use of these scoring systems can enhance the accuracy of prognosis prediction, thereby improving patient management and therapeutic outcomes.

In this study, we developed a machine learning-based prognostic model for predicting the 90-day mortality of patients with HBV-ACLF. We first performed differential analysis and univariate logistic regression to identify key clinical and laboratory characteristics associated with 90-day mortality in HBV-ACLF patients, revealing several potential risk and protective factors. Notably, variables such as age, liver failure stage (LF stage), hepatic encephalopathy (HE), and the ICG-R15 were found to be significant predictors of patient outcomes. Using these findings, we integrated 15 clinical features through 12 machine learning algorithms, including Least Absolute Shrinkage and Selection Operator (LASSO) and Random Forest (RF), to construct the most robust predictive model. The finalized model exhibited exceptional predictive accuracy, achieving an AUC of 0.99 and surpassing traditional scoring systems like MELD and CTP. Furthermore, we identified key prognostic features such as EHBF and LF stage through SHapley Additive exPlanations (SHAP) analysis. To facilitate clinical application, we developed an online tool that provides real-time prognostic predictions based on our model, enabling more accurate management and treatment strategies for HBV-ACLF patients.

## Methods

### Patient recruitment and data acquisition

**Figure 1:**
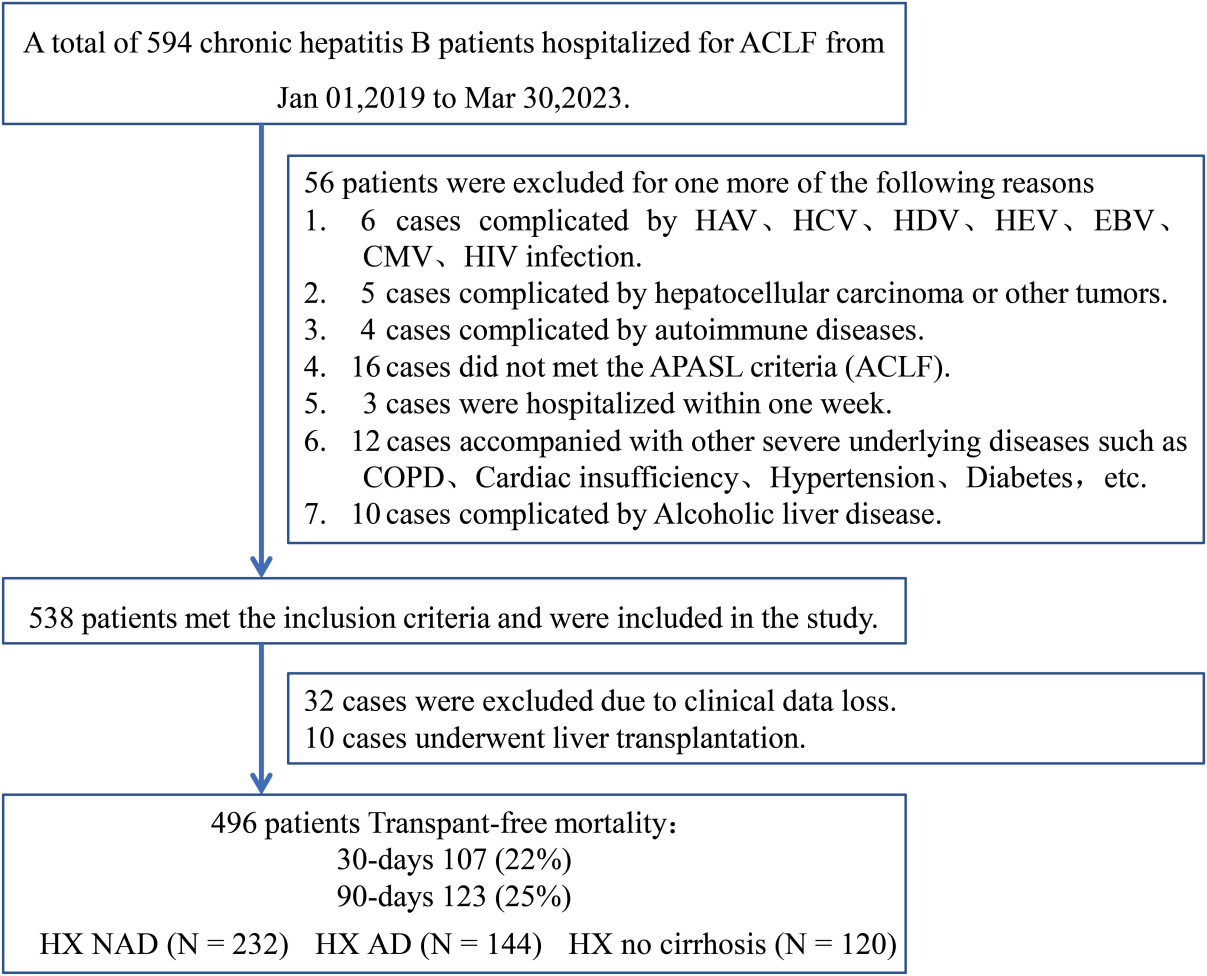
Flow chart of patient enrollment in this study.

In this study, a total of 496 patients with HBV-ACLF (HX cohort) were retrospectively recruited from the Center for Infectious Diseases, West China Hospital of Sichuan University, between January 1, 2019, and March 30, 2023. Within the HX cohort, patients were further stratified into predefined subgroups according to their clinical status at admission: the cirrhosis with non-acute decompensation group (HX-NAD, n=232), the cirrhosis with acute-decompensation group (HX-AD, n=144), and the non-cirrhotic group (HX–no cirrhosis, n=120). The external validation set (GZ cohort) comprised 52 inpatients from the Department of Infectious Diseases, Guizhou Provincial People’s Hospital, were enrolled between January 2017 and December 2021. Baseline demographics, complications, and laboratory measurements used as predictors were obtained within 7 days of admission (**Figure 1**).

### Criteria for inclusion and exclusion

During patient enrollment, the inclusion criteria were as follows: (1) a confirmed history of CHB infection with positive hepatitis B surface antigen (HBsAg) or detectable HBV DNA for more than six months; (2) acute liver injury characterized by jaundice with total bilirubin (TBIL) ≥5 mg/dL (85 μmol/L) and coagulation dysfunction, indicated by an international normalized ratio (INR) ≥1.5 or plasma thromboplastin antecedent (PTA) ≤40%; (3) clinical manifestations of ascites, HE, or both occurring within four weeks; and (4) liver reserve function assessed within 7 days of admission. Patients were excluded if they met any of the following conditions: (1) malignancies; (2) co-infections with other viruses such as hepatitis A virus (HAV), hepatitis C virus (HCV), hepatitis D virus (HDV), hepatitis E virus (HEV), Epstein-Barr virus (EBV), cytomegalovirus (CMV), or human immunodeficiency virus (HIV); (3) autoimmune hepatitis, alcoholic liver injury, or drug-induced liver injury; (4) pregnancy; (5) hospitalization duration less than seven days; or (6) incomplete clinical data or liver transplantation.

Data from 496 enrolled patients, including demographic characteristics, laboratory results, and complications (e.g., hyponatremia, hypokalemia, cirrhosis, ascites, HE, hepatorenal syndrome, and abdominal infections) were collected and analyzed. Liver reserve assessments were completed within 7 days after admission, with the first available measurement within this window used for analysis. Laboratory variables were extracted from routine tests obtained within the same 7-day window, using the first available value as the baseline for prediction. Complications were recorded if they occurred within 7 days after admission.

The study complied with the ethical guidelines of the Declaration of Helsinki and was approved by the Ethics Committee of West China Hospital of Sichuan University (approval number: 2023-2300).

### Diagnosis criteria

The diagnostic criteria for CHB adhered to the 2022 Chinese guidelines (You et al., 2023). ACLF was defined according to the Asia-Pacific Association for the Study of the Liver (APASL) 2019 consensus as an acute liver injury occurring in patients with pre-existing or ongoing chronic liver disease, characterized by jaundice (total bilirubin ≥ 5 mg/dL, approximately ≥ 85 μmol/L) and coagulopathy (INR ≥ 1.5), and complicated within 4 weeks by clinical decompensation manifested as ascites and/or HE (Sarin et al., 2019) Cirrhosis diagnosis was determined using imaging techniques, including ultrasonography, CT scans, and MRI, with typical findings such as liver shrinkage, splenomegaly, or ascites. In addition, ascites was diagnosed based on ultrasonography, CT, or MRI. (Tonon et al., 2024).

HE was diagnosed based on clinical manifestations and ammonia levels in the blood. Hyponatremia was diagnosed based on serum sodium concentration <135 mmol/L. Hepatorenal syndrome was diagnosed based on clinical manifestations and EGFR levels. Hypoproteinemia was defined as a serum albumin level <40g/L, and hypokalemia was defined as a serum potassium concentration <3.5 mmol/L. The LF stage should be determined based on the guidelines provided by the APASL (Sarin et al., 2019). Three existing prognostic scoring systems, including CTP, MELD, and LF stage were assessed in the patients based on data from hospitalization.

### Outcomes

The primary outcome was 90-day transplant-free mortality, defined as any death occurring within 90 days of follow-up without liver transplantation. For short, we used “90-day mortality” for analysis interpretation. The secondary outcome was 30-day transplant-free mortality, defined as any death occurring within 30 days of follow-up without liver transplantation.

### Machine learning algorithms

To construct the model, we employed a set of 12 machine learning techniques, including LASSO, Ridge, Stepglm, Extreme Gradient Boosting (XGBoost), RF, Elastic Net (Enet), Partial Least Squares Regression for Generalized Linear Models (plsRglm), Generalized Boosted Regression Modeling (GBM), Naive Bayes, Linear Discriminant Analysis (LDA), Generalized Linear Model Boosting (glmBoost), and Support Vector Machine (SVM). A comprehensive evaluation of 113 algorithmic combinations was conducted using the HX cohort training dataset. This process involved feature selection and model refinement within a tenfold cross-validation framework. The model’s performance was subsequently evaluated in subgroup analyses and in an external validation cohort (GZ cohort). Drawing from previous research, the best-performing model was identified as the one achieving the highest mean AUC across both the training and validation datasets (Qin et al., 2023).

### Statistical analysis

The baseline data analysis began by testing the normality of quantitative variables. Continuous variables with a normal distribution were represented as mean ± standard deviation (SD), and group comparisons were carried out using independent samples t-tests. For non-normally distributed data, the median (P25, P75) was reported, and comparisons between groups were performed using the Mann–Whitney U test. Categorical variables were summarized as frequencies (percentages, %), with statistical differences evaluated using chi-square tests. The diagnostic performance of the model was analyzed through ROC curve evaluation. LASSO and RF analyses were then applied to identify the final set of variables for inclusion in the model. A user-friendly web-based tool was developed using the Shiny R package. Statistical significance was defined as a two-tailed *P*-value of less than 0.05.

## Results

### Baseline patient demographics, clinical features, lab results, and mortality rates

A total of 496 individuals were included in this retrospective study. **Supplementary Table 1** provides a summary of their primary demographic and clinical characteristics, laboratory findings, and mortality outcomes. The median age of the participants was 46 years, and males comprised the majority, accounting for 87% of the cohort. Among the enrolled patients, 376 (76%) had cirrhosis, of whom 144 (29%) presented with acute decompensation, while 232 (47%) showed non-acute decompensation. During the follow-up period, a total of 123 deaths without transplantation were recorded. Of these, 107 deaths occurred within 30-day, corresponding to a 22% mortality rate, while 123 deaths occurred within 90-day, resulting in a 25% mortality rate.

### Differential analysis and univariate logistic regression of factors influencing 90-day mortality in HBV-ACLF patients

In order to examine the correlation between various clinical characteristics and the 90-day prognosis of HBV-ACLF patients, differential analysis and univariate logistic regression were conducted (**Supplementary Tables 2**, **3**). The results revealed that clinical characters such as age, ICG-R15, LF stage, HE, HE grade, ascites, ascites score, hepatorenal syndrome, TBIL, IBIL, NH3, PT, INR, Cr, NEUT, NEUT/LYM, CTP, and MELD were significantly elevated in the group of patients who died within 90-day, serving as potential risk factors for 90-day mortality in HBV-ACLF patients. On the contrary, ICG-K, EHBF, Na, PTA, and PLT exhibited significant decreases in the cohort of patients who experienced mortality within 90-day. Univariate analysis indicates that these factors could potentially serve as protective elements for the 90-day prognosis of HBV-ACLF patients. While cirrhosis did not show a significant association with the 90-day prognosis of ACLF patients, subgroup analysis indicated that the 90-day mortality rate was significantly increased in ACLF patients with acute decompensation of cirrhosis, highlighting acute decompensation of cirrhosis as a potential risk factor for patient prognosis.

### Development and validation of a novel HBV-ACLF prognostic model based on machine learning

**Figure 2:**
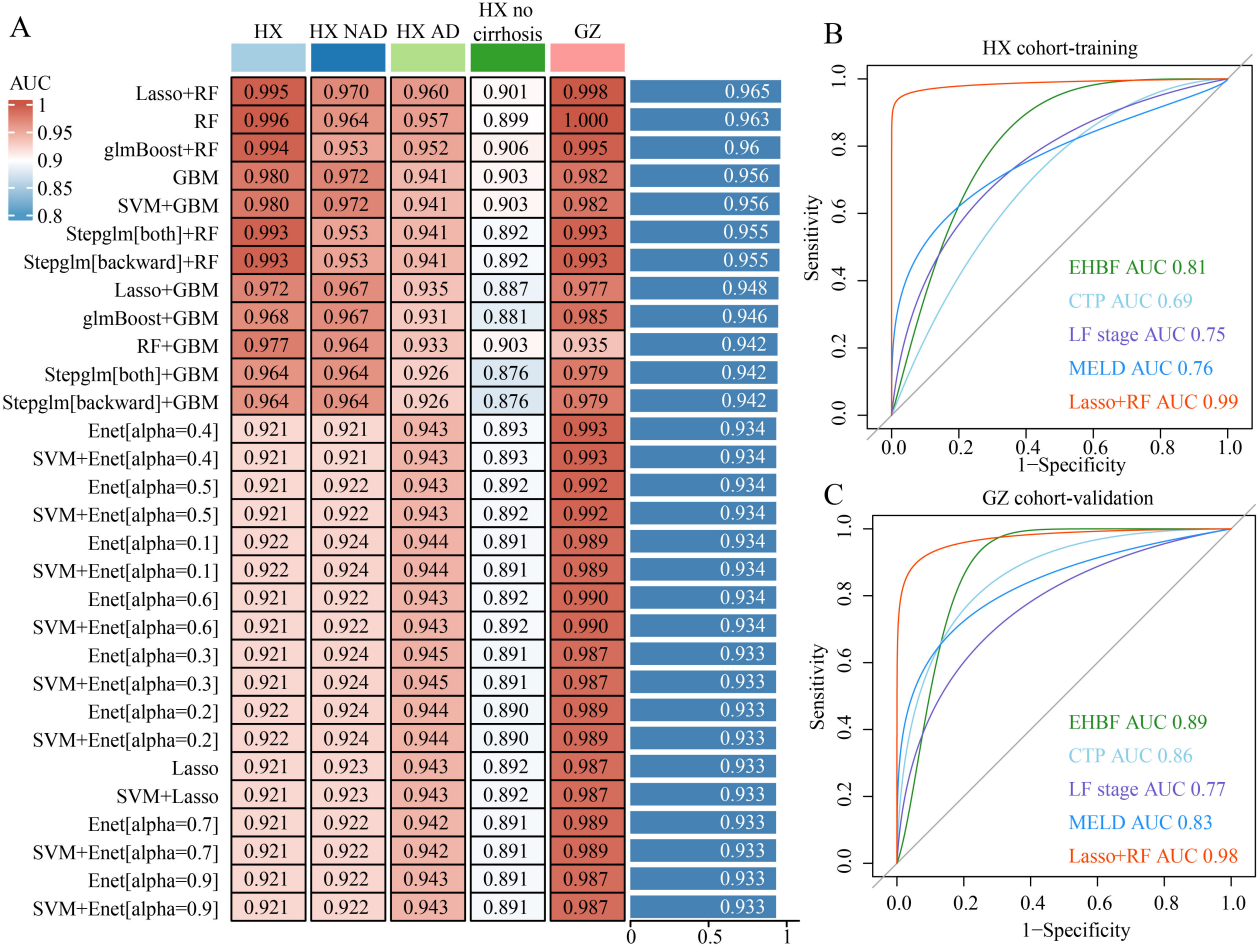
Diagnostic performance of our model. **(A)** 113 machine learning algorithm combinations evaluated via 10-fold cross-validation. **(B, C)** ROC curves of training cohort **(B)** and validation cohort **(C)**.

Twelve machine learning algorithms were integrated into a tenfold cross-validation framework to develop the most robust diagnostic model, leveraging 23 potential clinical risk factors to predict ACLF prognosis. This analysis was conducted using the training dataset (HX cohort), three subgroup datasets (HX-NAD, HX-AD, and HX–no cirrhosis), and an external validation dataset (GZ cohort). The final model, which exhibited the best performance, was developed by combining the LASSO and RF algorithms. The RF method identified 15 pivotal clinical features: EHBF, LF stage, hepatorenal syndrome, absolute neutrophil count, MELD, ICG-R15, Ascites score, TBIL, INR, PTA, age, HE stages, Na, NH3, and CTP (**Figure 2A**). Further ROC analyses demonstrated that the proposed model showed excellent discrimination for predicting 90-day outcomes in ACLF, with AUCs of 0.99 in the training cohort and 0.98 in the validation cohort. The model outperformed commonly used clinical indices, including EHBF, CTP score, LF stage, and MELD score (**Figures 2B, C**). Calibration analysis demonstrated good agreement between predicted and observed 90-day mortality in both the training and external validation cohorts (Hosmer-Lemeshow test, all P > 0.05, **Supplementary Figure 1**).

### XG-boost machine learning analysis identified key clinical prognostic features for HBV-ACLF

**Figure 3:**
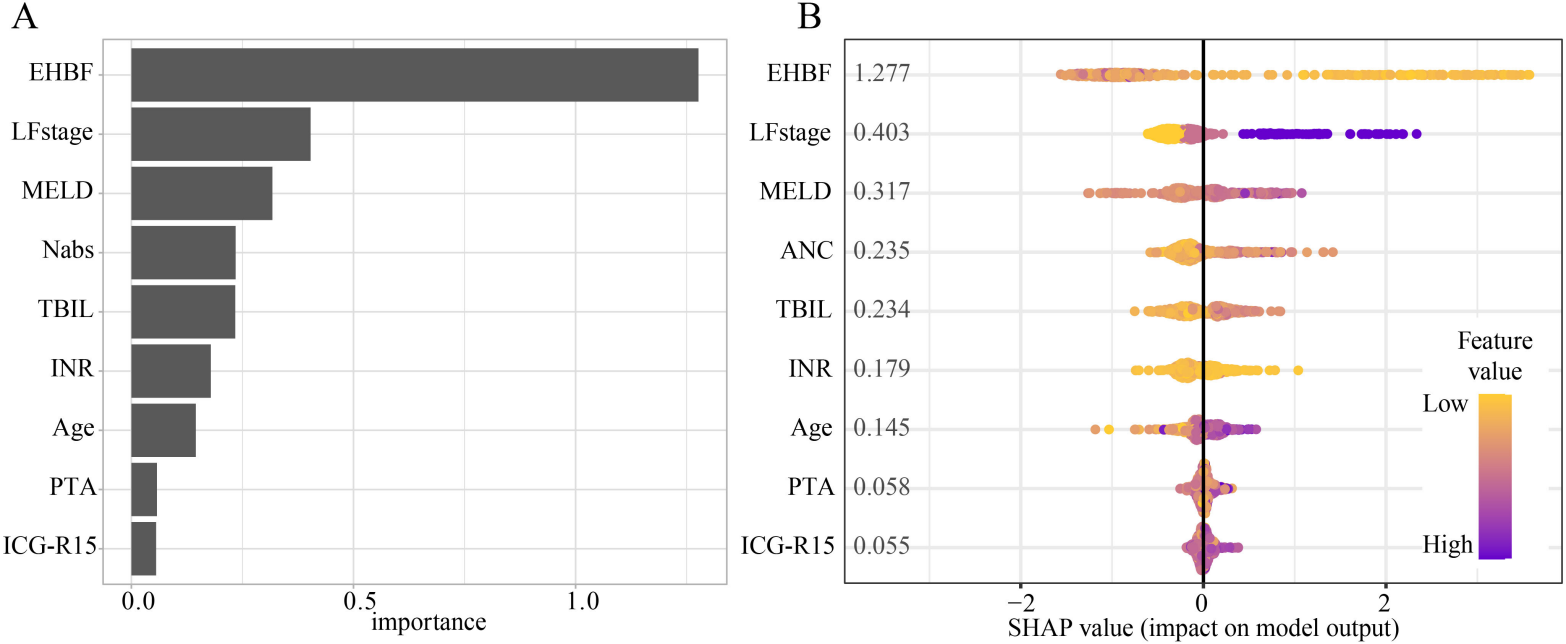
Importance of clinical factors to the 90-day mortality. **(A)** The importance of clinical factors to the 90-day mortality by SHAP value. **(B)** The top 9 (EHBF, LF stage, MELD, absolute neutrophil count, TBIL, INR, age, PTA, and ICG-R15) important regulators evaluated by the SHAP value.

The primary clinical characteristics associated with HBV-ACLF prognosis were identified using the XG-Boost algorithm combined with SHAP values. The analysis revealed that EHBF, LF stage, MELD score, absolute neutrophil count, TBIL, INR, age, PTA, and ICG-R15 were the top nine predictive features for HBV-ACLF prognosis (**Figure 3A**). Feature importance was further analyzed using SHAP values, with the ranking of these nine features illustrated in **Figure 3B**. Each point in **Figure 3B** represents the feature value of an individual patient, where the X-axis indicates the SHAP value and the color depth corresponds to the magnitude of the feature value. The ranking of variables was determined by summing the SHAP values across all samples. Among the identified features, EHBF and LF stage were found to be the top 2 critical prognostic factors for HBV-ACLF, as evidenced by their high feature importance scores and SHAP values in the XG-Boost analysis. Lower EHBF and higher LF stage were associated with an increased predicted 90-day mortality risk. The commonly used clinical parameter ICG-R15 was also identified as a critical prognostic factor, with higher ICG-R15 associated with an increased risk of 90-day mortality.

### The development and application of an online computing platform for the model

**Figure 4:**
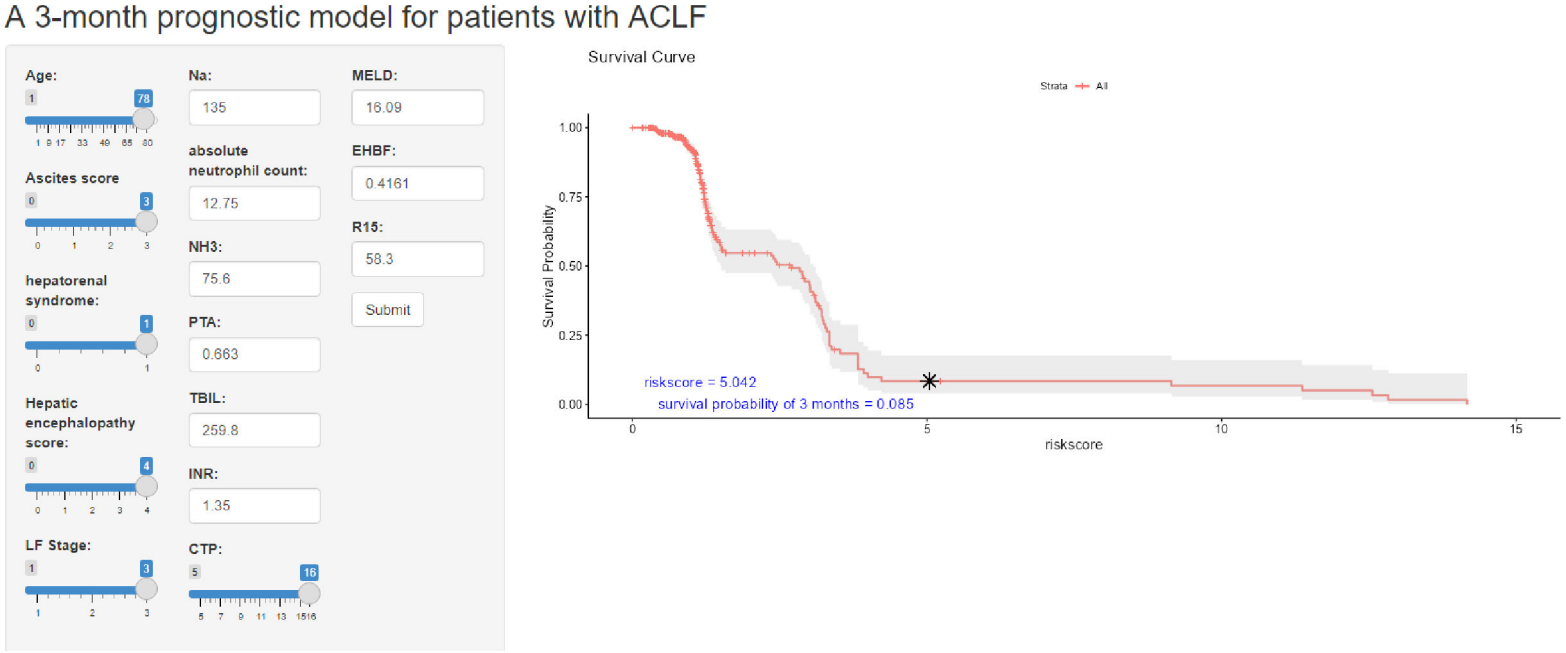
The online web tool for HBV-ACLF prognosis prediction, accessible at https://syx123.shinyapps.io/deploy_shiny/. The left panel allows input of clinical information, while the right panel displays the risk score and predicted 90-day mortality rate.

In this study, to facilitate clinical use, the risk scores for patients corresponding to the LASSO+RF model were calculated using the training set (HX cohort), and a survival curve was plotted showing a progressive decrease in survival rate with increasing risk scores. We categorized the 15 variables selected and compressed by LASSO into three groups: general clinical characteristics and complications, including age, ascites score, hepatorenal syndrome, HE score, LF stage, CTP and MELD; laboratory tests, including Na, absolute neutrophil count, NH3, PTA, TBIL, INR; and liver reserve, including, ICG-K, EHBF, and ICG-R15. Using these 15 variables, we ran a RF machine learning model and created an online platform at https://syx123.shinyapps.io/deploy_shiny/ (**Figure 4**). This platform will enable doctors and patients to utilize our model online, with corresponding risk scores for 90-day mortality rates indicated on the graph and accompanied by a textual prompt for interpretation.

## Discussion

Recent advancements have facilitated the development of various prognostic models for HBV-ACLF, providing improved tools for clinical evaluation and decision-making. For instance, a study (Wu et al., 2018) integrated the neutrophil-to-lymphocyte ratio, INR, age, and TBIL to construct a predictive model for 90-day mortality in HBV-ACLF patients. This model demonstrated superior predictive accuracy compared to traditional scoring systems such as MELD, MELD-Na, and CTP. Furthermore, a large multicenter prospective clinical study conducted in China (Pereira et al., 2012) analyzed clinical data from 1322 hospitalized patients with acutely decompensated cirrhosis and chronic hepatitis B-related liver injury across 13 liver disease centers. This study developed the COSSH-ACLFs model by combining key clinical parameters such as INR, Sequential Organ Failure Assessment (SOFA) score, age, TBIL, and other factors, offering a novel prognostic tool for HBV-ACLF. However, the etiology of HBV-ACLF varies regionally, with differences in diagnostic criteria and treatment protocols contributing to inconsistencies in prognostic assessments. Therefore, the development of more accurate and regionally adapted prognostic models for HBV-ACLF remains a critical priority.

Recent studies have made notable advancements in the role of liver reserve in predicting the prognosis of HBV-ACLF and liver cirrhosis (Lisotti et al., 2016; Wang et al., 2018; Moller et al., 2019; Chen et al., 2021; Zhou et al., 2022). Liver reserve, which reflects the liver’s remaining functional capacity to respond to physiological stress or injury, is a critical indicator for assessing the severity and prognosis of liver disease. Recent research has demonstrated the significant advantages of liver reserve testing in predicting postoperative liver failure and prognosis in liver cancer patients (Wang et al., 2018). In HBV-ACLF, the combination of EHBF and MELD score has also shown the highest prognostic predictive value (Chen et al., 2021). Therefore, this study systematically collected liver reserve information from patients, integrated detailed clinical characteristics, and employed machine learning to combine liver reserve testing with existing HBV-ACLF prognostic models, aiming to develop a new model capable of accurately predicting HBV-ACLF prognosis.

Machine learning holds broad prospects for the prognosis prediction of liver diseases, especially ACLF (Gary et al., 2023; Kalapala et al., 2023). In this study, we built a machine learning model that includes 15 variables, including age, ascites score, hepatorenal syndrome, HE score, and LF stage, CTP, MELD; laboratory tests, such as Na, absolute neutrophil count, NH3, PTA, TBIL, INR, and liver reserve factors, including ICG-R15 and EHBF. However, machine learning is often considered a “black box” technology, making it challenging for direct clinical application. To address this, we applied SHAP value to rank the importance of the variables, revealing that EHBF was the most significant predictor of prognosis in HBV-ACLF patients. This underscores the crucial role of liver reserve testing in predicting the prognosis of liver failure patients. Finally, to facilitate use by patients and clinicians, we developed an online computing platform for our model.

Despite the exceptionally high AUCs observed in both the training and external validation cohorts, the possibility of residual overfitting cannot be fully excluded. To mitigate this risk, we applied internal cross-validation, fixed the optimal probability threshold in the training cohort, and evaluated discrimination and classification metrics using an independent external cohort without threshold re-optimization. In addition, calibration analyses demonstrated good agreement between predicted and observed outcomes. Nevertheless, the external validation cohort was relatively small and derived from a single center, which may limit the precision of performance estimates and introduce center-specific effects (e.g., differences in case-mix, laboratory platforms, management pathways, and endpoint adjudication). Therefore, larger, multi-center and temporally external validations are warranted, and model recalibration may be required before broad clinical deployment.

## Conclusion

Indicators of hepatic reserve function, particularly EHBF and ICG-R15 play a pivotal role in predicting 90-day mortality outcomes in patients with HBV-related acute-on-chronic liver failure. Integrating liver functional reserve measures with clinical variables through machine learning approaches facilitates more precise and personalized prognostic assessments, thereby enhancing the ability to tailor clinical management strategies for this patient population.

## Data Availability

The original contributions presented in the study are included in the article/**Supplementary Material**. Further inquiries can be directed to the corresponding author.
