# Supplementary material for: Development and validation of a prognostic model for acute-on-chronic liver failure
Source: Front Cell Infect Microbiol. 2026 Feb 9;16:1759738. doi: 10.3389/fcimb.2026.1759738 (PMC12926412; doi:10.3389/fcimb.2026.1759738)
Supplement: Supplementary file 1 [file Supplementaryfile1.docx]

**Supplementary materials**


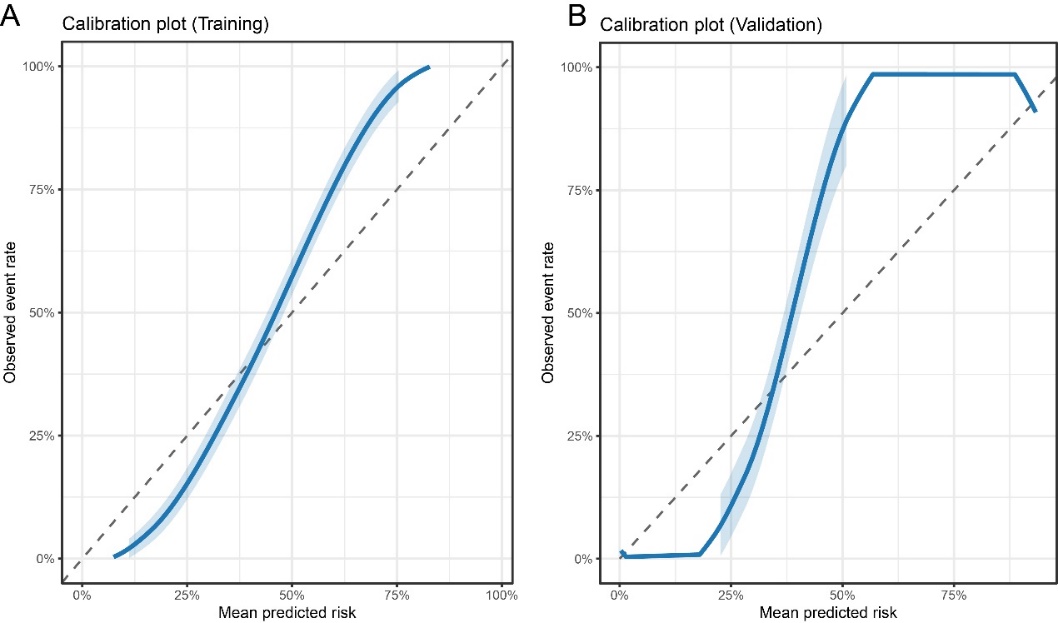


**Supplementary Figure 1. Calibration curves for model goodness-of-fit.** (A) Training cohort; (B) validation cohort.

Hosmer–Lemeshow (HL) goodness-of-fit test was also applied to evaluate agreement between predicted probabilities and observed outcomes. The HL test results were as follows: training cohort, χ² = 11.375, df = 8, *P* = 0.181; external validation cohort, χ² = 10.676, df = 8, *P* = 0.221. The findings indicated there was no statistical evidence of poor calibration or lack of fit in either cohort.
